# Effects of a locally administered risedronate/autogenous bone graft combination on bone healing in a critical-size rabbit defect model

**DOI:** 10.1186/s13018-023-03568-0

**Published:** 2023-02-04

**Authors:** Taha Özer, Vusala Guliyeva, Alper Aktaş, Emre Barış, Mert Ocak

**Affiliations:** 1 Department of Oral and Maxillofacial Surgery, Hacettepe University, Ankara, Türkiye; 2 Department of Oral Pathology, Gazi University, Ankara, Turkey; 3 Vocational School of Health, Ankara University, Ankara, Turkey

**Keywords:** Bone transplantation, Bone histomorphometry, Risedronate, x-ray microtomography, Immunohistochemistry

## Abstract

**Background:**

Risedronate is a bisphosphonate with poor oral absorption. An extremely hydrophilic molecule that has a high affinity for bone, risedronate also inhibits the farnesyl diphosphate synthase enzyme, inhibiting osteoclastic activity and reducing bone turnover and resorption. Autogenous bone grafts contain osteogenic cells and osteoinductive factors that are essential for bone regeneration and are therefore considered the gold standard. Thus, this study aimed to investigate the impact of local risedronate administered with autogenous bone grafts on the healing of defects in rabbit skulls using histological, histomorphometric, immunohistochemical, and three-dimensional radiological methods.

**Methods:**

Two 10-mm-diameter critical-size defects were created in 16 rabbits and filled with autogenous bone graft and autogenous bone graft + 5 mg risedronate in the control (C) and risedronate (RIS) groups, respectively. Residual graft, new bone, soft tissue areas, and bone volume were evaluated in the 4- and 8-week study groups.

**Results:**

There were no statistically significant differences in bone graft, new bone, or soft tissue area between the groups at 4 weeks (*p* > 0.05). At 8 weeks, the new bone area was significantly higher in the RIS group than in the C group (*p* < 0.05). The h scores obtained from sialoprotein and osteopontin did not differ significantly between the groups (*p* > 0.05). The radiologically measured total bone volume was significantly higher in the RIS group than in the C group at both time points (*p* < 0.05).

**Conclusions:**

In this study, risedronate enhanced the osteoconductive properties of autogenous bone grafts and rapidly created better-quality bone. This could improve future patient outcomes.

## Introduction

Graft operations are a common method used for replacing lost hard tissue, with approximately 2.2 million procedures performed annually worldwide. These operations involve numerous types of natural or synthetic grafts, particularly for the repair of defects resulting from atrophy, injury, congenital malformations, and tumor surgeries [1]. Among these, autogenous bone grafts are associated with higher morbidity rates, longer surgery times, and more postoperative complications as they require a second operation site. However, because they contain osteogenic cells and osteoinductive factors that are essential for bone regeneration, autogenous grafts remain the gold standard [2].

A derivative of bisphosphonate, risedronate, was first invented by Dr. Ann Geddes at Cincinnati Miami Valley Laboratories and later discovered again by a team of chemists led by Dr. Ray D’Alonzo and Dr. Kent Buckingham at the Norwich Eaton Procter and Gamble Laboratories [3]. Bisphosphonates are metabolically stable analogs of pyrophosphates and are formed by a bridging carbon atoms, replacing the oxygen atom in pyrophosphates that joins the two phosphate groups. This bridging carbon provides two sites for the side chains (R1 and R2). In risedronate, these side chains that are added to the central structure of bisphosphonates are the hydroxyl group (R1) and the pyridyl-methylene component (R2) [4, 5].

As with other bisphosphonates, risedronate has poor oral absorption, is a highly hydrophilic molecule, and has a high affinity for bones. It binds tightly to hydroxyapatite crystals in the bone, inhibits crystal growth and in vitro dissolution, and is rapidly transported to the bone in vivo, ultimately acting as an anti-resorptive drug. Risedronate also inhibits the farnesyl diphosphate synthase (FPPS) enzyme, inhibiting osteoclastic activity and leading to reduced bone turnover and resorption. Typically, risedronate has a slightly lower affinity for bone than other clinically used bisphosphonates such as alendronate and zoledronate [6]. However, risedronate is nearly as potent as zoledronate and minodronate as an inhibitor of FPPS target enzymes [7].

Previous research has shown risedronate to be more potent than alendronate in cell-based systems, to have a lower binding affinity to the bone matrix, and to be responsible for a relatively quicker stabilization of bone turnover inhibition when treatment is discontinued [4, 8].

Given these characteristics, this study aimed to investigate the impact of local risedronate administered with autogenous bone grafts on the healing of defect areas created in rabbit skulls using histological, histomorphometric, immunohistochemical, and three-dimensional radiological methods.

## Materials and methods

To investigate the impact of local risedronate administered with autogenous bone grafts on the healing of defect areas created in rabbit skulls, we used the following methods.

### Experimental model

This study protocol was performed in accordance with the National Institutes of Health ARRIVE guidelines for the care and use of laboratory animals and was independently reviewed and approved by the Animal Experiments Local Ethics Committee at XXX University, dated December 28, 2021, numbered 2021/10–02. We used 16 New Zealand rabbits (50% males and 50% females). One week before the study, the experimental animals were kept in cages under standardized room temperature, humidity, ventilation, and fluorescent light (12-h light/dark cycle) to adapt to environmental and climatic conditions. We then divided each experimental animal into two groups representing the right and left calvaria.

Each group was divided into two healing period time frames of 4 and 8 weeks for evaluation. We assigned the risedronate + autogenous bone graft (RIS) group to the left and the autogenous bone graft/control (C) group to the right calvaria for all experimental animals.

### Surgical procedure

**Fig. 1:**
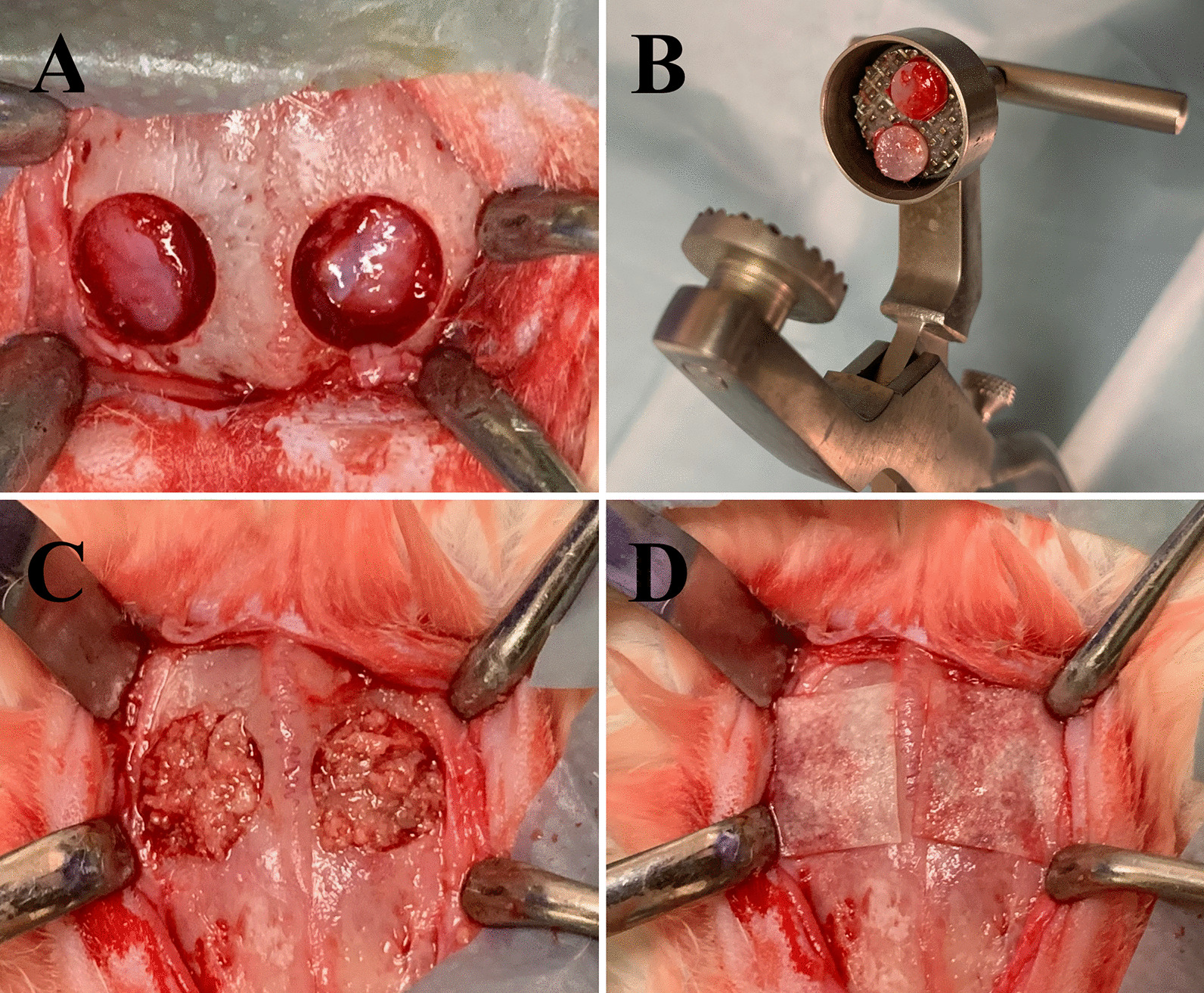
Protocol followed for bone grafting of calvarial bone defects in a rabbit model

For anesthesia, we administered ketamine hydrochloride (Alfamine, Alfasan, Netherlands) at a dose of 35 mg/kg and xylazine hydrochloride (Alfazyne, Alfasan, Netherlands) at 2.5 mg/kg intramuscularly to all experimental animals. The right and left cranial regions were shaved, and the operation site was cleaned using povidone-iodine (Batticon, Adeka, Turkey). We also applied a 1 ml local anesthetic solution (Ultracain D-S forte, Sanofi Aventis, Turkey) infiltratively to the operation site to control bleeding. We made an approximately 4-cm-full-thickness incision along the linea media on the midline of the calvaria that included the periosteum, using a No. 15 blade scalpel to expose the bone surface. Two bone osteotomies were then performed, one on each side of the linea media on the parietal bones, using a trephine drill with an outer diameter of 10 mm and an inner diameter of 9 mm under sterile saline cooling, with care taken not to damage the dura (Fig. 1A).

In the RIS group, disc-shaped cortical bones were removed to create the defect. They were then ground into small particles using a bone grinder (Fig. 1B), kept in local risedronate solution (5 mg risedronate-Sigma Aldrich-/1 ml saline) for 5 min, and then placed on the defect site (Fig. 1C). The grafted site was then covered with a collagen membrane that was rehydrated with a local risedronate solution (Fig. 1D).

In the C group, disc-shaped cortical bones were removed to create the defect, ground into small particles using a bone grinder (Fig. 1B), and used to fill the defect cavity, which was irrigated with sterile saline (Fig. 1C). The grafted site was then covered with a collagen membrane that was rehydrated with sterile saline (Fig. 1D).

Finally, we performed primary suturing on the cutaneous and subcutaneous tissues using a resorbable 16 mm 3/8 sharp 4.0 polyglactin suture (Coated Vicryl, Ethicon, Johnson & Johnson, Belgium). We then applied a wound spray (Opsite, Smith, & Nephew, Canada) to the sutured sites to prevent postoperative infections.

As a postoperative analgesic, we administered 1 mg/kg meloxicam (Maxicam X4; Sanovel, Turkey) and 2.5 mg/kg enrofloxacin (Baytril-K 5%; Bayer, USA) intramuscularly once a day for 5 days. Each experimental animal was housed in a separate cage with a 12-h light/dark cycle. In the cages, the ambient temperature was set to 22–24 °C and humidity to 55–70%. We provided the animals with adequate feed and water and regularly checked their wound sites.

### Tissue processing

Half of the experimental animals were sacrificed using a lethal dose of intramuscular xylazine HCl (30 mg/kg; Alfazyne, Alfasan, Netherlands) and ketamine HCl (70 mg/kg; Alfamine, Alfasan, Netherlands) at the end of the 4th week and half at the end of the 8th week. The examination site was removed from the cranium of each animal en bloc along with some surrounding intact bone tissue. We divided these samples into groups for each animal and fixed them in 10% buffered formaldehyde for 48 h.

### Radiological analysis

The samples were scanned using a micro-computed tomography scanner (Skyscan 1174, Skyscan, Kontich, Belgium) with a pixel size of 40 μm, X-ray tube voltage of 50 kV, current of 800 μA, and exposure time of 2300 ms. The X-ray projections were obtained at 0.70° intervals with a scanning angular rotation of 180°. Subsequent reconstruction of the raw data obtained from scanning was performed using NRecon software supplied by the manufacturer (NRecon version 1.6.9.4, Skyscan, Kontich, Belgium). The 8-bit grey images reconstructed using NRecon were imported into CTan software (version 1,13,5,1, Skycan, Kontich, Belgium), and the total bone volume and bone mineral density were then obtained from selected Regions of Interest.

### Histological analysis

Sixteen calvaria samples with two scaffolds were fixed with 10% formaldehyde for 48–72 h and then decalcified with DeCastroR solution (300 ml absolute ethanol, 50 g chloral hydrate, 670 ml distilled water, 30 ml 70% nitric acid) for 20 ± 2 days. Histomorphometric analyses were performed using specialized image analysis software (Leica Qwin plus V3; Leica Microsystems, Wetzlar, Germany). Five different images were obtained from each sample stained with hematoxylin and eosin at 100 × magnification. In each image, the areas of new bone trabeculae and soft tissue were calculated in μm^2^, and the mean values of the five images were retained.

Immunohistochemical staining was performed at the Thermo Scientific Shandon Sequenza Immunostaining Center (Thermo Shandon Limited, Runcorn, Cheshire, UK). The samples were taken from paraffin blocks and sliced into 4-μm-thick sections that were kept in an oven at 60 °C for 1 h, placed in xylol for 3 × 5 min, and deparaffinized. The slides were then subjected to a series of descending alcohol concentrations (100%, 96%, 80%, and 70%) and dehydrated. For antigen unmasking, we applied citrate buffer (PH6) with 1/10 dilution on a Thermo Scientific Shandon Sequenza immunostaining center (Thermo Shandon Limited, Runcorn, Cheshire, UK). In the immunohistochemical staining device, slides were attached to rack slots using cover plates. The cells were then washed with phosphate-buffered saline (PBS) for 5 min.

Endogenous peroxidase activity was blocked with 3% hydrogen peroxide (TA-125-HP; Thermo Scientific) for 10 min. Protein block (TA-125-PBQ Thermo Scientific) was applied to the tissue sections, and primary antibodies were incubated using anti-osteopontin (ab63856 Abcam) and anti-bone sialoprotein antibodies (ab52128 Abcam) at 4 °C for one night. Washing was subsequently performed for 5 min with PBS.

The amplifier Quanto (TL-125-QPB, Thermo Scientific) was incubated for 20 min, and the HRP Polymer Quanto (TL-125-QPH, Thermo Scientific) for 30 min. The cells were washed with PBS at each step. To detect positive cells, staining was performed using DAB chromogen (TA-125-HD, Thermo Scientific). For floor staining, hematoxylin (HHS32; Sigma) was applied for 30 s. The samples were then washed with distilled water for 2 × 1 min, passed through a series of ascending alcohol concentrations (70%, 80%, 96%, and 100%), placed in xylol for 2 × 1 min and capped with Entellan (C1795 Merck).

Immunohistochemical analysis was then performed under a light microscope at × 100 magnification. Staining of the extracellular matrix and cell cytoplasm within the defect site was considered positive for osteopontin (OSP) and bone sialoprotein (BSP). In these samples, staining intensity and staining extent were evaluated together, and numerical data were obtained by adhering to the h-score index as shown below (Table 1). The h-score was obtained by multiplying the staining intensity by the staining extent.

**Table 1:** The h score index showing the staining intensity and the extent of staining

Staining intensity		Staining extent	
0	No staining	0	No staining
1	Slight staining	1	% 0–25
2	Mild staining	2	% 26–50
3	Severe staining	3	% 51–75
		4	% 76–100

*h*: staining intensity X staining extent

### Statistical analysis

Data analysis was performed using SPSS 21.0 statistical package software (IBM Corp., Armonk, NY, US). The results are presented as mean, median, and standard deviation. Intergroup comparisons were made using the Mann–Whitney U test and intragroup by the Wilcoxon test, comparing 4th-week and 8th-week data. For the type 1 error level (alpha), values that were smaller than 0.05 were considered statistically significant.

## Results

**Fig. 2:**
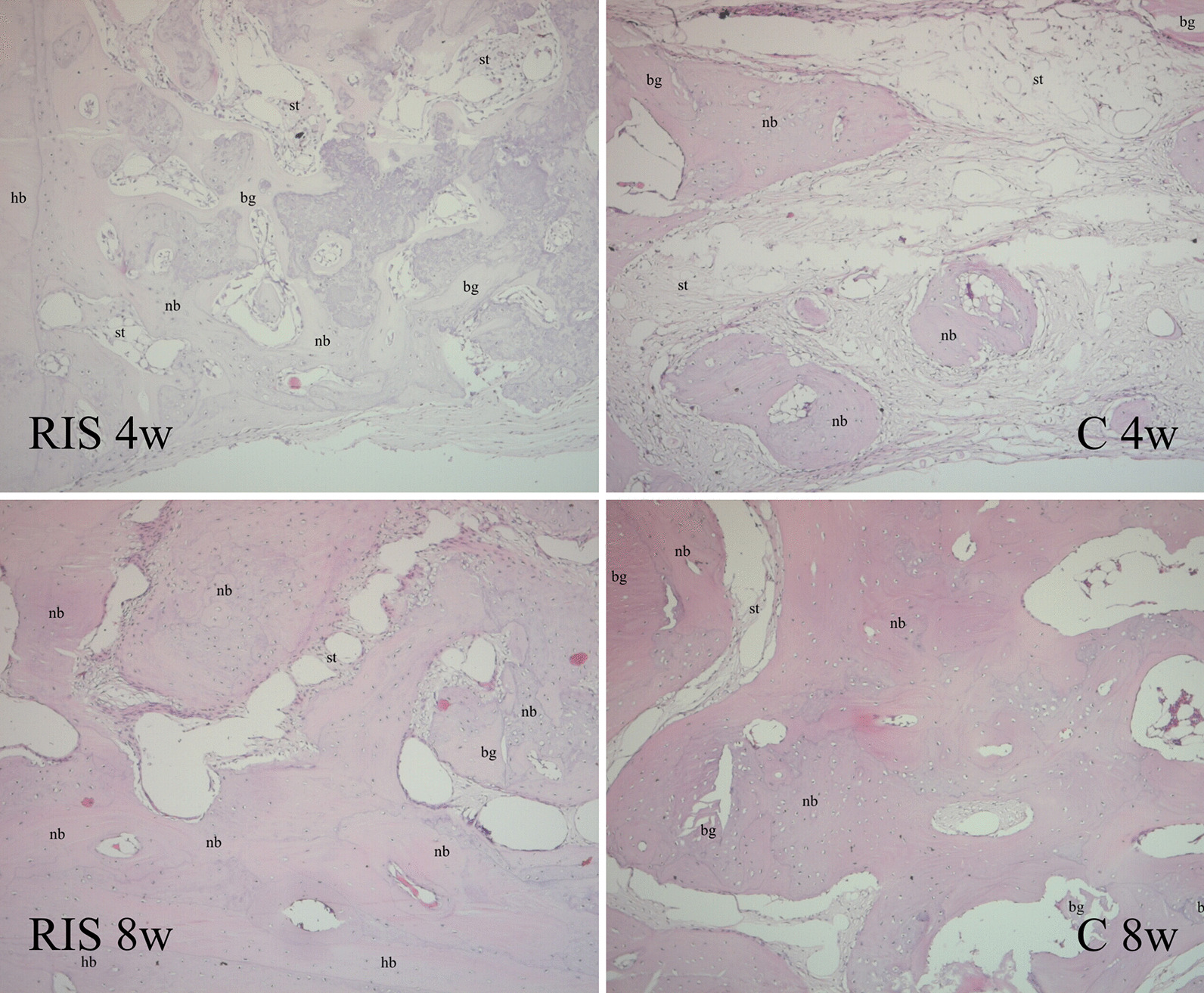
Histological findings at 4 and 8 weeks after surgery for calvarial bone defects in a rabbit model (C group, autogenous bone grafting only; RIS group, risedronate with autogenous bone grafting; nb, new bone trabeculae; bg, bone graft material; st, soft tissue; hb, host bone; hematoxylin and eosin, × 100 magnification)

**Fig. 3:**
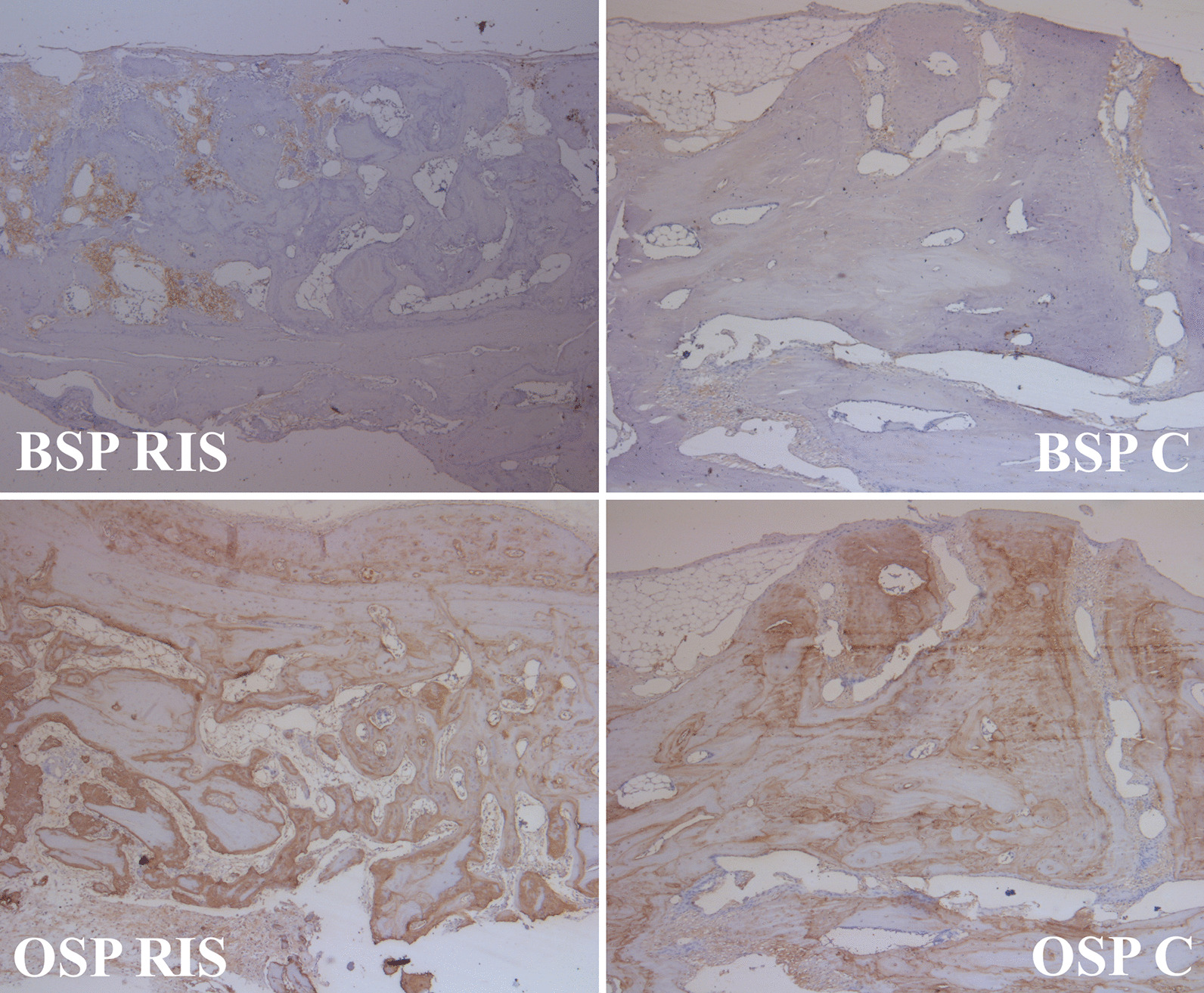
Immunohistochemical findings at 8 weeks after surgery for calvarial bone defects in a rabbit model (BSP, bone sialoprotein; OSP, osteopontin; C group, autogenous bone grafting only; RIS group, risedronate with autogenous bone grafting; × 40 magnification)

In the histological preparations of the 4-week samples, we observed that the defect sites consisted of new bone trabeculae and loose collagenized connective tissue in nearly all samples. New bone production appeared to have migrated from the edges of the defect toward the center, and its trabeculae formed around the graft particles, most of which were shaped as trabeculae in the periphery of the defect due to the reactivation of the injured periosteum. In the 8th-week preparations, the overall histological appearance was similar to that of the corresponding 4th-week samples, with significantly more bone trabeculae in the RIS group and fewer graft particles remaining between these trabeculae than in the 4th-week samples (Figs. 2 and 3).

**Fig. 4:**
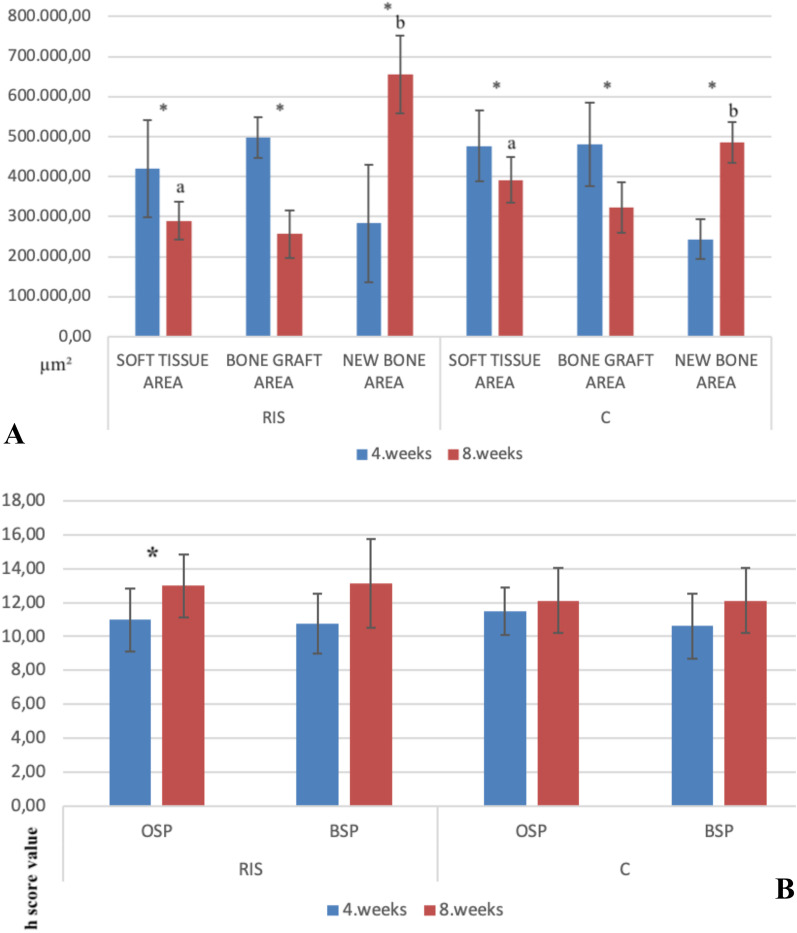
**A** New bone, bone graft, and soft tissue area measured by histomorphometry at 4 and 8 weeks after surgery for calvarial bone defects in a rabbit model. Data are presented as the mean ± standard deviation. (C group, autogenous bone grafting only; RIS group, risedronate with autogenous bone grafting; *, significantly different from values obtained 8 weeks after surgery; a, b, significantly different from values obtained in the other group, black line indicate the standard deviation.) **B** Comparison of h score values obtained from the immunohistochemical staining at 4 and 8 weeks after surgery. Data are presented as the mean ± standard deviation (C group, autogenous bone grafting only; RIS group, risedronate with autogenous bone grafting; *, significantly different from values obtained 8 weeks after surgery; black line indicates the standard deviation)

In the 4th-week results of histomorphometric data, we found no statistically significant difference between the groups. Regarding the 8th-week results, the new bone area was significantly higher in the RIS group than in the C group (*p* < 0.05). Moreover, the soft tissue area was significantly larger in the C group than in the RIS group (*p* < 0.05). The differences in histomorphometric parameters from the 4th week to the 8th week were statistically significant across both groups. In both groups, the new bone area was found to have increased significantly from the 4th to the 8th week (*p* < 0.05), while the soft tissue area and bone graft area decreased significantly over the same period (Fig. 4A).

The h scores obtained from BSP and OSP did not differ significantly between the groups in the 4th or 8th week (*p* > 0.05). In the RIS group, the OSP increased significantly from the 4th to the 8th week (*p* < 0.05), although there was no statistically significant increase in the OSP in the C group (*p* > 0.05). The BSP did not display a statistically significant difference from the 4th week to the 8th week in either group (*p* > 0.05) (Fig. 4B).

**Fig. 5:**
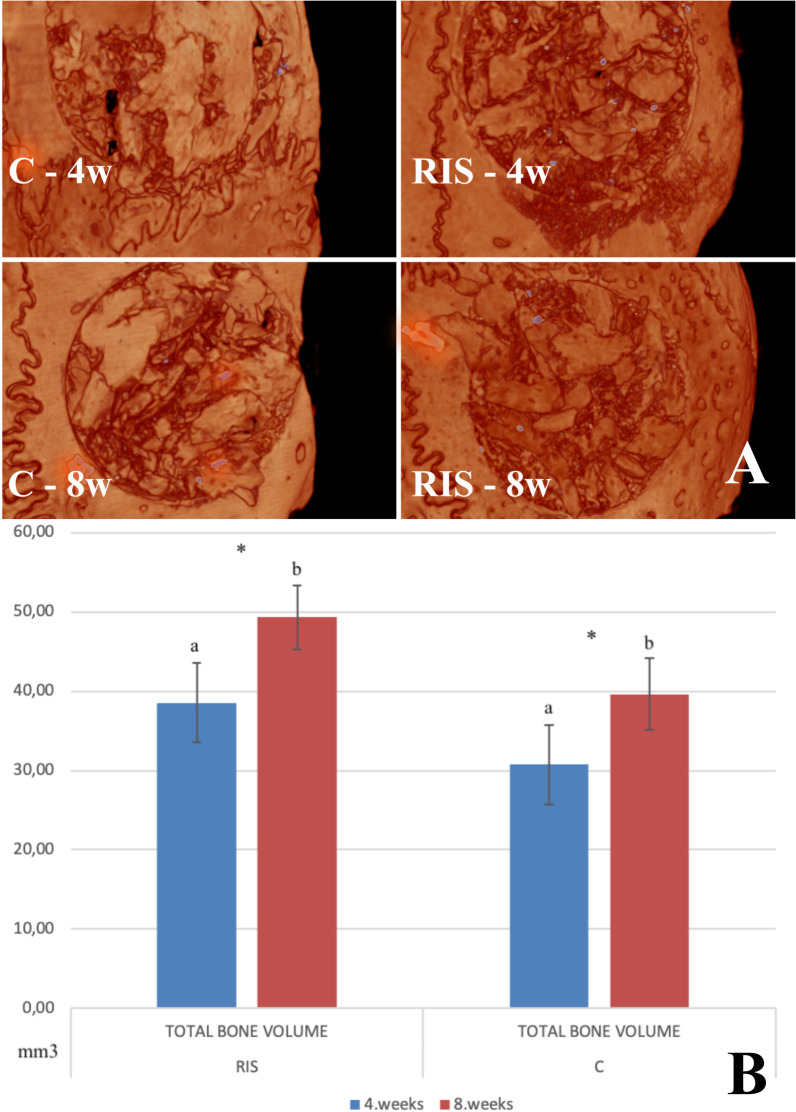
**A** Micro-computed tomography (CT) images of all groups at 4 weeks and 8 weeks. **B** Comparison of values for the total bone volume derived from radiographs obtained 4 and 8 weeks after surgery. Data are presented as the mean ± standard deviation (C group, autogenous bone grafting only; RIS group, risedronate with autogenous bone grafting; *, significantly different from values obtained 8 weeks after surgery; a, b, significantly different from values obtained in the other group, black line indicates the standard deviation)

The total bone volume was significantly higher in the RIS group than in the C group at both weeks 4th and 8th weeks (*p* < 0.05). In addition, the increase in bone volume from the 4th to the 8th week was statistically significant in both groups (*p* < 0.05). Finally, no statistically significant difference was observed in bone mineral density (Fig. 5).

## Discussion

In this study, we enhanced the osteoconductive properties of autogenous bone grafts and created better quality bones rapidly using locally applied risedronate.

Recently, the rapid ossification of grafts in bone surgery has become a popular subject of study. To this end, researchers have investigated the use of various chemical and biological molecules such as statins, stem cells, and bisphosphonates under different methods. Bisphosphonates are typically used orally in low doses for the treatment of osteoporosis; however, they are also used parenterally and in high doses in some types of cancer to prevent bone metastasis. However, this use causes a series of complications owing to a high level of suppression of osteoclastic activity resulting in deterioration of bone turnover [9]. While their current use has been successful in conditions ranging from postmenopausal osteoporosis to Paget’s disease and malignant hypercalcemia, bisphosphonates used in the long term have recently been associated with systemic side effects including renal toxicity, osteonecrosis of the jawbone, atypical femur fractures, and hypocalcemia [10].

Bisphosphonate-related osteonecrosis of the jaw (BRONJ) with its new name medication-related osteonecrosis of the jaw (MRONJ) is the most serious side effect of bisphosphonates. It defined as exposed bone or bone that can be probed through any fistula in the maxillofacial region that persists for more than eight weeks in patients without any radiation therapy who have been treated with powerful antiresorptive or angiogenesis inhibitor drugs. The most important risk factors for osteonecrosis are the type of bisphosphonate and the dosage. Especially in patients treated with intravenous bisphosphonates, the risk of osteonecrosis is much higher than the use of oral bisphosphonates. In addition, conditions such as cancer diagnosis, history of trauma or surgery, poor oral hygiene, dental infections, long-term corticosteroid therapy, suppression of the immune system, vascular insufficiency, and old-age increase the probability of this complication. Typical symptoms of the cases are pain, soft tissue edema, infection, tooth mobility, halitosis, purulent discharge and exposed necrotic bone. Symptoms may develop spontaneously or appear after oral surgical procedures. MRONJ occurs only in the jawbones, due to the continuous relationship of the jawbones with the oral flora, through teeth or dentures. In addition, bisphosphonates are stored more in bones with high metabolic rate such as jaw bones. High doses of bisphosphonates cause intracellular calcium deposition in both osteoblasts and osteoclasts, producing a cytotoxic effect. As a result, the bone regeneration mechanism is disrupted and the required remodeling cannot occur. Decreased bone regeneration increases the risk of avascular necrosis. When osteoclastic activity begins in the bone, cytokines and growth factors are secreted to mature the new bone matrix. With the decrease in bone resorption, apposition also begins to decrease; bone repair metabolism, growth and development capacity and quality are impaired. In necrosis due to trauma, periodontal disease, periradicular lesion or dental procedure; osteoclasts cannot be activated to remove necrotic bone [11, 12].

Numerous efforts to take advantage of the local effects of bisphosphonates while preventing side effects have been reported, and evidence shows that the concentration level can be maintained at the targeted site [13, 14]. For example, Toker et al. reported no significant difference between local and systemic applications of bisphosphonate in defect sites in rat skulls [15]. Similarly, Küçük et al. found no significant difference between local and systemic applications of bisphosphonates in a rabbit distraction model [16]. Numerous researchers have also shown the positive effects of drugs belonging to the bisphosphonate group in cases where bone healing is prominent given the local suppression of osteoclastic activity. Hence, the administration of bisphosphonates by this local method with minimal systemic side effects has become much more popular. The current study supports the relevant literature in this regard by concluding that the local use of risedronate, a potent bisphosphonate, increases both the total bone and newly formed bone volume.

Risedronate has a strong anti-resorptive effect without strong inhibition of mineralization [17]. Çetinkaya et al. investigated the daily use of low- (0.1 mg/kg) and high-dose (1 mg/kg) oral risedronate on alveolar bone loss in mice with periodontitis, concluding that short-term low-dose risedronate use inhibited bone resorption, whereas high-dose long-term use led to deteriorated bone formation and angiogenesis [18]. Aghayan et al. used risedronate in a 2% gel form (2 g) in rabbits and evaluated its impact on bone healing using histomorphometric analysis and found that the experimental group displayed more ossification and more osteoblast cells at the end of two months compared to the control group [19].

Unlike Aghayan et al., we administered risedronate in its pure form and at a dose of 5 mg/ml. This has some significance for demonstrating the impact of risedronate and isolating those of additional biological materials. Khajuria et al. used biodegradable chitosan as a carrier for risedronate in the treatment of periodontitis in a rat model. Despite their positive findings, the potential share of chitosan in this study remains controversial [20]. In the current study, we used collagen membranes and autogenous graft materials as carriers of risedronate. Using this method, we maintained autogenous grafts and collagen membranes in a solution prepared with pure risedronate for 5 min, thus excluding the biological effects of other materials used as carriers.

Guided bone regeneration (GBR) was first described in 1959 to prevent the invasion of rapidly growing tissues into the bone regeneration zone, thereby optimizing bone healing and ensuring that relevant growth factors remain in the regeneration zone. This technique has been used safely by numerous surgeons in vertical and horizontal bone augmentation and peripheral nerve surgery for many years. Today, surgeons continue to perform successful bone reconstructions using various GBR methods and materials [21, 22].

With successful clinical use for over 100 years, autogenous bone grafts remain the gold standard and first choice in bone graft selection because of the osteogenic capacity of living cells. While some disadvantages such as donor site morbidity and limited availability exist, they are gradually being eliminated owing to recently defined autogenous bone harvesting methods [23]. We selected autogenous bone grafts for this study because of the osteogenic, osteoinductive, and osteoconductive properties provided by their osteoprogenitor cells, growth factors, and connective tissue proper matrices, respectively.

The barriers used for GBR must have certain properties including biocompatibility, cell permeability, and space protection. In addition, resorbable membranes should not prevent bone regeneration through minimal tissue reactions during resorption. Numerous barrier membranes have been reported including polytetrafluoroethylene, expanded polytetrafluoroethylene, collagen, freeze-dried dura mater, and titanium foils [24]. In this study, we chose to use collagen membranes because of their high biocompatibility and low antigenicity. These membranes also served as carriers for risedronate, eliminating the need for another biological agent. To the best of our knowledge, our study is the first where collagen membranes have been used with risedronate in the literature and are therefore highly valuable in evaluating the pure impact of local risedronate.

The suitable sites for creating experimental defects in rabbits for research purposes include the mandible, calvaria, femur, tibia, fibula, and radius. We chose the calvaria as the defect site because of its ease of transportation and application, similar ossification patterns with maxillofacial region bones, and ease of comparison with similar previous research. There have been many successful trials using rabbit skulls as surgical sites. Furthermore, by choosing this region, which offers a sufficient surgical area, we ensured the elimination of individual differences, forming both groups on the same anatomical region of the same animal.

A critical-sized bone defect is defined as the smallest bone wound in an animal that cannot spontaneously heal with bone filling throughout its life without using osteopromotive materials. This defect is determined based on the size of each bone in each animal species. For the skull bone of rabbits, the area of this type of defect has been determined as two 10-mm-diameter circles according to multiple studies. Previous studies have created 10-mm-diameter, bilateral, calvarial defects and allowed them to heal spontaneously, reporting less than 20% ossification [25–27]. In relevant research, using critical-sized defects is valuable and necessary to indicate the contribution of grafts or chemicals to bone healing in these defects, which cannot heal spontaneously. In the current study, we used the critical-sized bone defects determined for the rabbit skulls.

The rate of bone metabolism in experimental rabbits is three times faster than that in humans. Given that humans complete ossification within six months after surgery, the corresponding process in rabbits could be interpreted as a period of eight weeks [28]. Previous research has observed angiogenesis in rabbits at the end of four weeks, indicating bone healing. Miloro et al. investigated various time frames to observe early and late bone healing, specifically examining the 2nd, 4th, 8th, and 12th weeks. The authors highlighted that the 4th week was the most appropriate time for evaluating ossification in the early period and the 8th week in the late period [28, 29]. Therefore, given this information, we chose to sacrifice the experimental rabbits at the 4th and 8th weeks to examine early and late ossification, respectively.

Newly formed bone volume measurements and soft tissue evaluations were performed using two-dimensional histomorphometric and three-dimensional micro-CT analyses, as histomorphometric analyses alone have the limitation of being two-dimensional and involve time-consuming sample preparation and formation of artificial tissue [30, 31]. Thus, current approaches tend to include micro-CT.

In addition, immunohistochemical analysis is a key method for identifying important proteins in the extracellular matrix that plays a role in bone formation. In this study, we evaluated the OSP and BSP and used h-scores to evaluate the density and prevalence of proteins in the samples, which were calculated by modifying the h-score formulation in Karataş et al. and were analyzed semi-quantitatively [32]. According to our results, there were no significant differences between the groups. Nevertheless, the increases in BSP and OSP among the 8th-week samples were noteworthy compared to those in the 4th-week samples. The fact that these proteins increased over time in both groups was interpreted to indicate strong future ossification.

In our RIS group, the newly formed bone volume was significantly higher among the 8th-week samples, which is explained by the fact that risedronate induces new bone formation in the late period. The effect of risedronate during the early period was only demonstrated by the total bone volume measured radiologically. This may also stem from the early anti-resorptive effect of risedronate on autogenous bone grafts. This change, without alteration of the existing graft area, was supported by a significant reduction in soft tissue area over time. When applied locally with grafts, bisphosphonates are expected to maintain the graft at the defect site without resorption and to increase the total bone area. In this study, we observed that risedronate had an anti-resorptive effect on the graft in the early period and induced new bone formation in the late period.

This study had some limitations, including the dosage and method of administration of the drug, possible drug diffusion between defects, and the possibility of systemic effects by the locally administered drug. Future research should determine the most appropriate dose in this context.

## Conclusions

In conclusion, we were able to achieve the goal of enhancing the osteoconductive properties of autogenous bone grafts and creating better-quality bones rapidly using locally applied risedronate, which may improve patient outcomes in the future.

## Data Availability

There were no additional data used for your results beyond what is presented in this paper.

## Abbreviations

BRONJ: Bisphosphonate-related osteonecrosis of the jaw
BSP: Bone sialoprotein
C: Control
FPPS: Farnesyl diphosphate synthase
GBR: Guided bone regeneration
MRONJ: Medication-related osteonecrosis of the jaw
OSP: Osteopontin
RIS: Risedronate
